# Particle-Driven Effects at the Bacteria Interface: A Nanosilver Investigation of Particle Shape and Dose Metric

**DOI:** 10.1021/acsami.3c00144

**Published:** 2023-08-15

**Authors:** Lisa M. Stabryla, Paige J. Moncure, Jill E. Millstone, Leanne M. Gilbertson

**Affiliations:** † Department of Civil and Environmental Engineering, University of Pittsburgh, 3700 O’Hara Street, Pittsburgh, Pennsylvania 15261, United States; ‡ Department of Chemistry, University of Pittsburgh, 219 Parkman Avenue, Pittsburgh, Pennsylvania 15260, United States; § Department of Chemical and Petroleum Engineering, University of Pittsburgh, 3700 O’Hara Street, Pittsburgh, Pennsylvania 15261, United States; ∥ Department of Mechanical Engineering and Materials Science, University of Pittsburgh, 3700 O’Hara Street, Pittsburgh, Pennsylvania 15261, United States

**Keywords:** antimicrobial activity, surface area, crystal facet, ion release, controlled NP synthesis, poly(vinylpyrrolidone), particle concentration, EC_50_

## Abstract

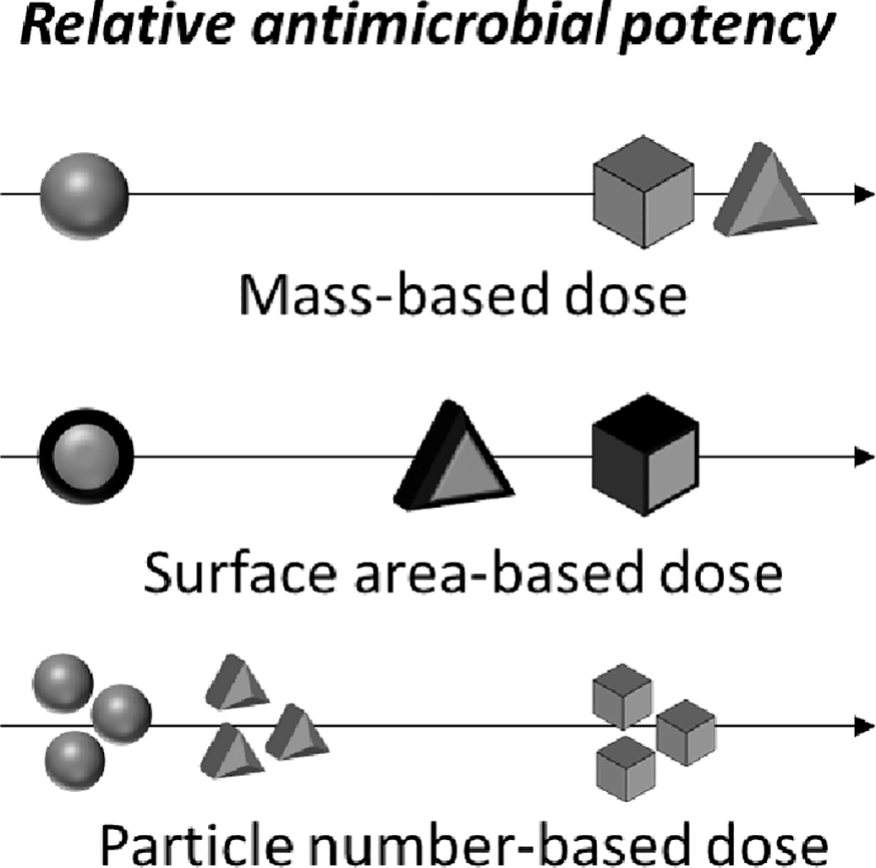

Design criteria for controlling engineered nanomaterial (ENM) antimicrobial performance will enable advances in medical, food production, processing and preservation, and water treatment applications. In pursuit of this goal, better resolution of how specific ENM properties, such as nanoparticle shape, influence antimicrobial activity is needed. This study probes the antimicrobial activity toward a model Gram-negative bacterium, *Escherichia coli* (*E. coli*), that results from interfacial interactions with differently shaped silver nanoparticles (AgNPs): cube-, disc-, and pseudospherical-AgNPs. The EC_50_ value (i.e., the concentration of AgNPs that inactivates 50% of the microbial population) for each shape is identified and presented as a function of mass, surface area, and particle number. Further, shifts in relative potency are identified from the associated dose–response curves (e.g., shifts left, to lower concentrations, indicate greater potency). When using a mass-based dose metric, the disc-AgNPs present the highest antimicrobial activity of the three shapes (EC_50_: 2.39 ± 0.26 μg/mL for discs, 2.99 ± 0.96 μg/mL for cubes, 116.33 ± 6.43 μg/mL for pseudospheres). When surface area and particle number are used as dose metrics, the cube-AgNPs possess the highest antimicrobial activity (EC_50_-surface area: 4.70 × 10^–5^ ± 1.51 × 10^–5^ m^2^/mL, EC_50_-particle: 5.97 × 10^9^ ± 1.92 × 10^9^ particles/mL), such that the relative trend in potency becomes cubes > discs > pseudospheres and cubes ≫ discs ⩾ pseudospheres, respectively. The results reveal that the antimicrobial potency of disc-AgNPs is sensitive to the dose metric, significantly decreasing in potency (∼5–30×) upon conversion from a mass-based concentration to surface area and particle number and influencing the conclusions drawn. The shift in relative particle potency highlights the importance of investigating various dose metrics within the experimental design and signals different particle parameters influencing shape-based antimicrobial activity. To probe shape-dependent behavior, we use a unique empirical approach where the physical and chemical properties (ligand chemistry, surface charge) of the AgNP shapes are carefully controlled, and total available surface area is equivalent across shapes as made through modifications to particle size and concentration. The results herein suggest that surface area alone does not drive antimicrobial activity as the different AgNP shapes at equivalent particle surface area yield significantly different magnitudes of antimicrobial activity (i.e., 100% inactivation for cube-AgNPs, <25% inactivation for disc- and pseudospherical-AgNPs). Further, the particle shapes studied possess different crystal facets, illuminating their potential influence on differentiating interactions between the particle surface and the microbe. Whereas surface area may partly contribute to antimicrobial activity in certain ENM shapes (i.e., disc-AgNPs in relation to the pseudospherical-AgNPs), the different magnitudes of antimicrobial activity across shape provide insight into the likely role of other particle-specific factors, such as crystal facets, driving the antimicrobial activity of other shapes (i.e., cube-AgNPs).

## Introduction

There is a growing demand to develop design criteria for antimicrobial engineered nanomaterials (ENMs), particularly ionizing, inorganic metal and metal–oxide nanoparticles, which represent a large fraction of nanomaterials used in the pharmaceutical and healthcare sector.^1−3^ Further resolution of relationships between tunable nanoparticle properties (i.e., size, shape, and surface chemistry) and antimicrobial outcomes is required to control functional performance for the intended application.^2,4−7^ It is well-established that the particle surface plays a critical role in the magnitude and mechanisms of antimicrobial activity, yet the distinction between particle-specific mechanisms, independent from and synergistic with ionization, remains unresolved.^4,6−8^ Our previous work demonstrated the role of silver nanoparticle (AgNP) size (<10 nm) and surface chemistry (positive-charge) as parameters that could elicit particle-specific effects independent from and synergistic with the release of Ag(I) ions.^7^ The link between particle-specific antimicrobial activity and NP shape is particularly challenging to isolate because the generation of NP shapes requires specific particle surface chemistries that could influence antimicrobial activity on its own.^9−16^ Further, different NP shapes have intrinsic differences in surface facet populations^13,14,17,18^ and NP shape is often dynamic in solution (where high energy facets typically are replaced by lower energy surfaces via a variety of growth and etching mechanisms).^11,13−15,19^ Thus, distinguishing shape effects independent of consequential particle properties (e.g., size, surface chemistry, surface area) and experimental system influences (e.g., model organism, growth medium) often results in conflicting conclusions across studies.

Interactions between AgNPs and microorganisms that precede an adverse outcome necessarily occur at the interface of the NP and cell surface. While the influences of AgNP shape on surface reactivity (e.g., exposed crystal facets,^17,18,20−22^ dissolution,^23−25^ and reactive oxygen species production^26−28^) have been studied, linking particle-specific shape properties to antimicrobial effects has not been established and is necessary to enable intentional particle design. Several shape-focused AgNP and Ag_2_O studies conclude the presence of differential antimicrobial activity arising from NP shape distinctions,^18,23,24,26,29−34^ while others conclude the absence of shape-based effects (i.e., shape does not influence bacterial response)^35−37^). Additionally, there is no consensus for a relative measure of antimicrobial activity by shape. For example, triangular plates and prisms,^18,23−25,29,31,32^ cubes, pseudospheres, and hexagons^25,26,29,30,33,34^ have all been identified as imparting the highest antimicrobial activity across different comparative studies. Distilling which shape is most efficacious and under what conditions (or for which organisms) is not possible from the available data for a variety of reasons (e.g., different measured endpoints, different experimental systems, different mass-based exposures). Further, studies that investigate the influence of AgNP shape on antimicrobial activity do not reach consensus on whether the induced differential outcomes are ion-, particle-, or synergistically driven. Those studies suggesting a measured biological response of different AgNP shapes arises from ion-driven effects^23,37^ indicate that particle shape enhances ion release through (i) differential surface reactivity (arising from the exposed surface facets) and/or (ii) particle surface area.^18,23−25,30,38^ Yet, the influence of particle shape on dissolution kinetics is not conclusive since these studies do not routinely and comprehensively monitor ion release.^18,23−25,30,38^ Only one study evaluated antimicrobial activity with scaled ion controls, where Ag(I) ions are dosed at concentrations equivalent to those quantified through ion release studies in the associated AgNP system.^37^ There are also studies suggesting that the particles themselves induce the observed differential shape effects via enhanced membrane association, production of reactive oxygen species (ROS), and physical interaction with the cell driven by increased surface reactivity of certain facets and increased cell contact area.^17,26,32,33,39^ Similar particle-driven effects have been found for other ionizing ENMs, such as Cu_2_O.^6,28^ While the complexity of the nanoparticle system underlies some of the existing mechanistic ambiguity (e.g., nanoparticle shape can increase binding to the bacterial cell membrane and cell wall constituents as well as enhance the dissolution rate of silver atoms from the nanoparticle surface via oxidation), a non-trivial component arises from inconsistencies in experimental designs across studies.^4,7^ Critical components of the experimental designs include:

(i) the extent to which other NP physiochemical properties (i.e., size, surface chemistry, surface area) are controlled across the NP shapes being studied (i.e., often size and surface chemistry are not held constant and change simultaneously along with shape),^4,7,18,23,32,37,39,40^
(ii) experimental conditions such as the model organism and growth medium used, the choice of assay, and measured toxicity endpoint (i.e., various organisms and media are used^4,7,24,30,36,41−45^ and these different experimental conditions influence particle dynamics, such as aggregation and dissolution, which are known to influence the measured outcome^7,41^ and alter the conclusions of shape-based antimicrobial activity),
(iii) the wide range in selected mass-based AgNP concentrations and other biologically relevant dose metrics,^46,47^
(iv) inclusion of particle stability monitoring, particularly in the experimental system,^7^ and
(v) inclusion of a Ag(I) ion control to distinguish between particle-specific and ion-driven effects.^7^

Attributing the observed differences in antimicrobial activity to ionization is complex, and studies often do not include scaled ion controls necessary to attribute behavior to the Ag(I) ion component nor monitor Ag(I) ion release under the same conditions as the biological experiments to support these claims, both of which critically influence the conclusions drawn.^7^ All of the abovementioned complications can serve as powerful modalities for controlling antimicrobial activity, such that isolating shape-derived, nanoparticle-specific antimicrobial behaviors and their mechanistic origin(s) will inform greater functionality in ionizing metal and metal oxide antimicrobials.

Notably, particle surface area, total available surface area, or effective cell contact area has been identified as contributors to observed differential antimicrobial activity arising from nanoparticle shape.^18,23,26,31^ Since only molecules on the particle surface are in direct contact with the cell, differences in surface area based on particle shape are anticipated to impact antimicrobial activity. Particle surface area can influence ion release and directly relates to available surface interaction with the cell, thus influencing the amount of physical contact that occurs to impart downstream chemical (e.g., ROS production) and physical (e.g., membrane disruption) mechanisms of antimicrobial activity.^33,48−50^ Given that surface-related modes of action likely contribute to shape-based differences in antimicrobial activity and that surface areas vary widely across particle systems, dose metrics such as geometric surface area or particle number (i.e., the number concentration of particles) can aid in evaluating relative shape-based ENM antimicrobial activity. Further, differing results would reveal the biological relevance of surface area as a dose metric for NP shapes compared with the conventional mass-based dose metric. While most antimicrobial studies of NP shapes are based on mass, the relevance of other dose metrics, such as geometric surface area and particle number, is acknowledged in numerous cell-based *in vitro* and animal-based *in vivo* toxicity studies.^48,51−58^ In fact, using mass as the sole dose metric is identified as supporting false conclusions that smaller particles are inherently more toxic than larger particles, the size-dependence of which vanishes when surface area is used as the dose metric.^59,60^ Thus, studies that solely compare mass-based activity miss more nuanced conclusions influenced by surface area or other particle variables that are not accounted for when measured biological endpoints are normalized to surface area or other particle variables.^23,25,26,30,31,34^

While current studies offer valuable insight into shape-dependent AgNP antimicrobial activity, there remains an opportunity to identify (i) the intrinsic shape-driven material properties that govern the observed behavior, (ii) the role that the AgNP (versus the Ag(I) ion) plays in shape-dependent inactivation, and (iii) the underlying physical and chemical mechanisms imparted by particle and ion system components. In this work, we prepare and comprehensively characterize three AgNP shapes: cube-, disc-, and pseudospherical-AgNPs. These particles were carefully synthesized, with surface charge and surface area held constant while systematically varying shape. We then uniquely evaluate shape-based AgNP antimicrobial activity by comparing mass-based activity with surface area- and particle number-based activity. Dissolution and stability of the three AgNP shapes is also monitored in the experimental system and, when combined with particle characterization, informs relationships between specific particle physicochemical properties and antimicrobial activity. We report the influence of AgNP shape on *Escherichia coli* (*E. coli*) antimicrobial activity and isolate both surface area and crystal facets as nanoparticle-specific factors driving the observed shape-dependent antimicrobial activity independent from Ag(I) ion release. Specifically, we observe that different AgNP shapes at equivalent particle surface area yield significantly different magnitudes of antimicrobial activity using both theoretical (by converting the measured toxicity outcomes to available surface area) and empirical approaches. This suggests that surface area alone does not govern antimicrobial activity, and that while in certain shapes (i.e., disc-AgNPs), surface area may partly contribute to antimicrobial activity, in other shapes (i.e., cube-AgNPs), particle variables such as crystal facets are likely the dominant contributor. Further, our study highlights the importance of using surface area and particle number as biologically relevant dose metrics for predicting and assessing shape-based ENM antimicrobial activity.

## Materials and Methods

### Chemicals

Silver nitrate (AgNO_3_, 99.9999%), tannic acid (purissimum grade), sodium citrate tribasic dehydrate (citrate, ≥99%), sodium hydrosulfide (NaHS), poly(vinylpyrrolidone) (PVP, average MW = 29,000 Da and 55,000 Da), sodium borohydride (NaBH_4_, ≥99%), hydrogen peroxide (H_2_O_2_, 30 wt %), ethylene glycol (EG), nitric acid (HNO_3_, >99.999%, trace metal basis), and a silver standard for ICP (Fluka, TraceCERT 1001 ± 2 mg/L Ag in HNO_3_) were purchased from Sigma-Aldrich (St. Louis, MO). Hydrochloric acid (HCl, >99.999%, trace metal basis), sodium chloride (NaCl, 99.6%), Luria-Bertani broth (LB, Miller), BD Bacto dehydrated agar, and glycerol were purchased from Fisher Scientific (Waltham, MA). All reagents were used as received unless otherwise indicated. All chemicals were dissolved in deionized (DI) water obtained from a Milli-Q ultrapure water purification system. NANOpure water (Thermo Scientific, ≥18.2 MΩ cm) was used in the preparation of all solutions associated with AgNP synthesis. Before use, all glassware and Teflon-coated stir bars were washed with aqua regia (3:1 ratio of concentrated HCl and HNO_3_ by volume) and rinsed thoroughly with water. Caution: aqua regia is highly toxic and corrosive and requires proper personal protective equipment. Aqua regia should be handled in a fume hood only.

### AgNP Characterization

PVP-capped pseudospherical-, cube-, and disc-AgNPs were synthesized according to previously reported procedures, which are detailed in the Supporting Information.^14,61,62^ The three AgNP shapes were confirmed after synthesis by ultraviolet–visible–near infrared (UV–vis–NIR) spectroscopy using a Cary 5000 spectrophotometer (Agilent, Inc.). The spectrum baseline was corrected with respect to the H_2_O spectrum. To evaluate the size and shape of the AgNPs, NP size and shape distributions were determined by transmission electron microscopy (TEM). An aliquot was obtained from the stock solution and diluted with H_2_O prior to being drop cast (∼8 μL) onto a carbon type A 200 mesh copper TEM grid (Ted Pella, Inc.). Samples were allowed to slowly air dry and then were dried under vacuum overnight before characterization with a Hitachi H-9500 environmental TEM at 300 kV and attached Gatan Orius camera (NanoScale Fabrication and Characterization Facility, Petersen Institute of NanoScience and Engineering, Pittsburgh, PA). NP size and shape distributions were determined from measuring at least 300 NPs (ImageJ 1.52a, National Institutes of Health, USA) in multiple images collected from various areas of the grid. Finally, AgNP stock concentrations and Ag(I) ion release profiles were characterized with inductively coupled plasma mass spectrometry (ICP-MS) and optimal emission spectrometry (ICP-OES), the methods for which are outlined in the Supporting Information.

### Zeta Potential and Particle Stability Characterization

Dynamic light scattering (DLS) and electrophoretic light scattering (ELS) were further used to characterize each shape’s hydrodynamic diameter (HDD), particle size distribution, polydispersity index (PDI), zeta potential, and colloidal stability in ultrapure water using a Litesizer 500 (Anton Paar, Inc). For DLS analysis, AgNP solutions were placed in disposable plastic cuvettes (Fisherbrand). Spectra were averaged over 30 scans at 10 s measurements. For ELS analysis, AgNP solutions were placed in a polycarbonate Omega cuvette (Anton Paar). Spectra were averaged over 20 scans. For both types of analyses, at least three replicates were included.

### Bacterial Strains and Cultivation

The bacterial strain used in this work was the wild-type *E. coli* K-12 MG1655(Seq) strain (CGSC #7740) obtained from the Coli Genetic Stock Center at Yale University. *E. coli* is both a clinically and environmentally relevant pathogen (part of the ESKAPE pathogens) and is the predominant Gram-negative bacteria to cause extraintestinal illness in humans and can cause urinary tract infection, pneumonia, and meningitis, among others. *E. coli* is a major cause of nosocomial infections, including catheter-associated UTIs and ventilator-associated pneumonia. *E. coli* can also be found in soil, on vegetables, and in water as well as in undercooked meats. Microorganism stock solutions were prepared in LB broth supplemented with 25% glycerol and stored at −80 °C. Microorganisms were cultured overnight in LB broth at 37 °C and 150 rpm. Overnight cultures were re-inoculated in fresh medium and grown to exponential phase (optical density, OD = 1; 10^9^ colony forming units (CFU)/mL), after which they were washed three times with 0.9% NaCl at 13,000*g* for 1 min and diluted 100-fold in 0.9% NaCl to obtain approximately 10^7^ CFU/mL.

### Antimicrobial Activity of AgNPs

The antimicrobial activity of the three AgNP shapes was evaluated using standard planktonic half-maximum effective concentration (EC_50_) measurements. The EC_50_ concentration is a universal toxicological endpoint enabling comparison across materials. In this study, the EC_50_ was characterized by a reduction of bacterial cell viability determined by a decrease in CFU. CFU counts are a direct and accurate measure of viable cells, especially in the case of *E. coli*, which can be cultured in the lab. These experiments were carried out in 96-well flat bottom microplates (Corning Costar) and done in biological and technical triplicates (*n* = 9) at each particle concentration. A dispersion of AgNPs was serially diluted in H_2_O and inoculated with 50 μL of *E. coli* (10^7^ CFU/mL) in 0.9% NaCl so that the total volume of each well was 100 μL. The final tested silver concentrations were 200, 100, 50, 25, 12.5, 6.3, 3.1, 1.6, and 0.8 μg/mL for the cube- and disc-AgNPs and 1250, 625, 300, 150, 75, 37, 18, 9, 4, 2, and 1 μg/mL for the pseudospherical-AgNPs. A negative control (no AgNPs added) treatment was created by adding 50 μL of sterile DI water. Cells were incubated at 37 °C for 3 h with medium linear shaking (∼500 cpm) in a Microplate Reader (Synergy HTX Multi-Mode, BioTek). After the 3 h contact time, the bacteria–AgNP suspensions were diluted (1:10) in Eppendorf tubes and vortexed, and 50 μL of each suspension was spread on a LB agar plate and incubated overnight at 37 °C for CFU enumeration (i.e., the parameter used to determine the EC_50_). The mean plate count and standard deviation for three biological replicates (*n* = 9) are reported.

### Effective Concentration Calculation

The EC_50_ was determined in OriginPro 8.5.1 software using a sigmoidal fit of the dose–response function with eq 1:
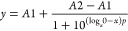
1where *A*1 is the bottom asymptote, *A*2 is the top asymptote, log_*x*_0 is the center, and p is the hill slope, and EC_50_ is given by eq 2
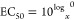
2

### Antimicrobial Activity of AgNPs as a Function of Surface Area

To investigate the effects of surface area, the antimicrobial activity of the three AgNP shapes was considered as a function of surface area both via calculation (i.e., theoretically) and empirically in the lab. First, the toxicity data, as EC_50_, was converted to particle number (i.e., the number concentration of particles) and surface area using ideal geometry approximations (see Tables 1 and 2 for conversion factors used in these calculations). Example calculations are outlined in the Supporting Information. Next, inactivation experiments were carried out at particle concentrations where the total amount of surface area available in each shape system was determined to be equal. Here, all concentrations were scaled to have the same available surface area present and chosen at intermediary concentrations in order to distinguish effects between the shapes, while avoiding high concentration-induced aggregation, complete bacterial inactivation, and other consequential influences on cytotoxicity. For these reasons, the selected concentrations were based on the EC_50_ of the disc-AgNPs such that the total surface area in the system was 1.40 × 10^–4^ m^2^ mL^–1^. The final silver concentrations used were 2.6, 9.0, and 11.4 μg/mL for the disc-, cube-, and pseudospherical-AgNPs, respectively, confirmed by both UV–vis–NIR and ICP-MS/OES. Inactivation of *E. coli* exposed to these concentrations in 96-well microplates was evaluated by plating biological and technical triplicates (*n* = 9) at different time points over 3 h (*t* = 0, 0.25, 0.5, 1, 2, and 3 h). The mean plate count and standard deviation for three biological replicates (*n* = 9) is reported.

**Table 1:** Summary of the Geometries, Volumes, and Surface Areas of the AgNP Shapes^a^

	cubes	discs	pseudospheres
radius, *r*, nm^b^	n/a	n/a	45.9 ± 9.8 nm
edge length, *a*, nm^b^	36.2 ± 6.8 nm	19.1 ± 5.0 nm	n/a
thickness, *t*, nm^b^	n/a	5.5 ± 1.3 nm	n/a
hydrodynamic diameter, nm^c^	135.35 ± 15.89 nm	27.81 ± 2.77 nm	104.43 ± 1.38 nm
zeta potential, mV^c^	–25.91 ± 0.65 mV	–31.10 ± 1.60 mV	–38.93 ± 1.45 mV
polydispersity index^c^	26.58 ± 1.93%	30.02 ± 1.55%	19.42 ± 0.65%
theoretical crystal facets	anisotropic, 100% facets	anisotropic, variable % {100} and {111} facets	amorphous, 63.4% {100} and 36.6% {111} facets
volume equation	a^3^	πr^2^t	4/3πr^3^
estimated volume per particle, nm^3^	47,437.9 ± 27,202 nm^3^	1575.1 ± 827 nm^3^	50,607.7 ± 33,150 nm^3^
surface area equation	6a^2^	2πrt + 2πr^2^	4πr^2^
estimated surface area per particle, nm^2^	7862.6 ± 2858.3 nm^2^	902.6 ± 386.9 nm^2^	6615.4 ± 2830.2 nm^2^
surface area/volume per particle, nm^–1^	0.17 ± 0.03 nm^–1^	0.57 ± 0.06 nm^–1^	0.13 ± 0.03 nm^–1^

a Mean values for particle radii, edge lengths, and thickness for the different shapes as well as estimated per particle volume and surface area, approximated using ideal geometries and average edge lengths.

b Determined via TEM (see methods for details on establishing average radius and edge length values).

c Determined via DLS and ELS in ultrapure water using at least three replicates (for DLS, 30 scans per replicate, and for ELS, 20 scans per replicate).

**Table 2:** EC_50_ of Each Shape Converted to Surface Area and Particle Number with Lower and Upper 95% CI Endpoints Included in Parentheses

	cubes	discs	pseudospheres
mass-based EC_50_, μg/mL	2.99 ± 0.96	2.39 ± 0.26	116.33 ± 6.43
	(1.07–4.91)	(1.87–2.91)	(103.47–129.19)
	trend in relative potency: discs ⩾ cubes ≫ pseudospheres		
	relative magnitude: discs are ∼1.5× more potent than cubes and ∼50× more potent than pseudospheres		
EC_50 _ converted to surface area, m^2^/mL	4.70 × 10^–5^ ± 1.51 × 10^–5^	1.30 × 10^–4^ ± 1.41 × 10^–5^	1.44 × 10^–3^ ± 7.94 × 10^–5^
	(1.68 × 10^–5^−7.72 × 10^–5^)	(1.02 × 10^–4^−1.58 × 10^–4^)	(1.28 × 10^–3^−1.60 × 10^–3^)
	trend in relative potency: cubes > discs^a^ > pseudospheres		
	relative magnitude: discs are ∼10× more potent than pseudospheres and cubes are ∼3× more potent than discs		
EC_50 _ converted to particle number, particles/mL	5.97 × 10^9^ ± 1.92 × 10^9^	1.55 × 10^11^ ± 1.63 × 10^11^	2.18 × 10^11^ ± 1.17 × 10^11^
	(2.13 × 10^9^−9.81 × 10^9^)	(1.22 × 10^11^−1.88 × 10^11^)	(1.95 × 10^11^−2.41 × 10^11^)
	trend in relative potency: cubes ≫ discs^b^ ⩾ pseudospheres		
	relative magnitude: discs are ∼1.5× more potent than pseudospheres and cubes are ∼30× more potent than discs		

a Discs are 5× relatively less potent when considering surface area compared to on a mass basis.

b Discs are 30× relatively less potent when considering particle number compared to on a mass basis.

### Statistical Treatment of Data

As stated above, the EC_50_ (μg/mL) values of the various particle shapes were determined from the sigmoidal dose–response curves using a sigmoidal fit. These experiments were tested statistically by assessing if the 95% confidence intervals for the best fit EC_50_ parameter (μ) overlapped. If the 95% confidence intervals overlapped, the two dose–response curves were considered not statistically different. Approximate 95% confidence interval end points for μ were calculated using eq 3
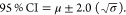
3

GraphPad Prism version 9.5.1 (La Jolla, California, USA) was used to assess the difference in bacterial inactivation by the three AgNP shapes as well as their ion release profiles. One-way ANOVA with Tukey’s multiple comparison test was used to compare the three AgNP shapes at each time point. The significance level is 95%, *i.e.*, *P* values smaller than 0.05 are considered statistically significant.

## Results and Discussion

### Characterization of AgNP Suite

**Figure 1:**
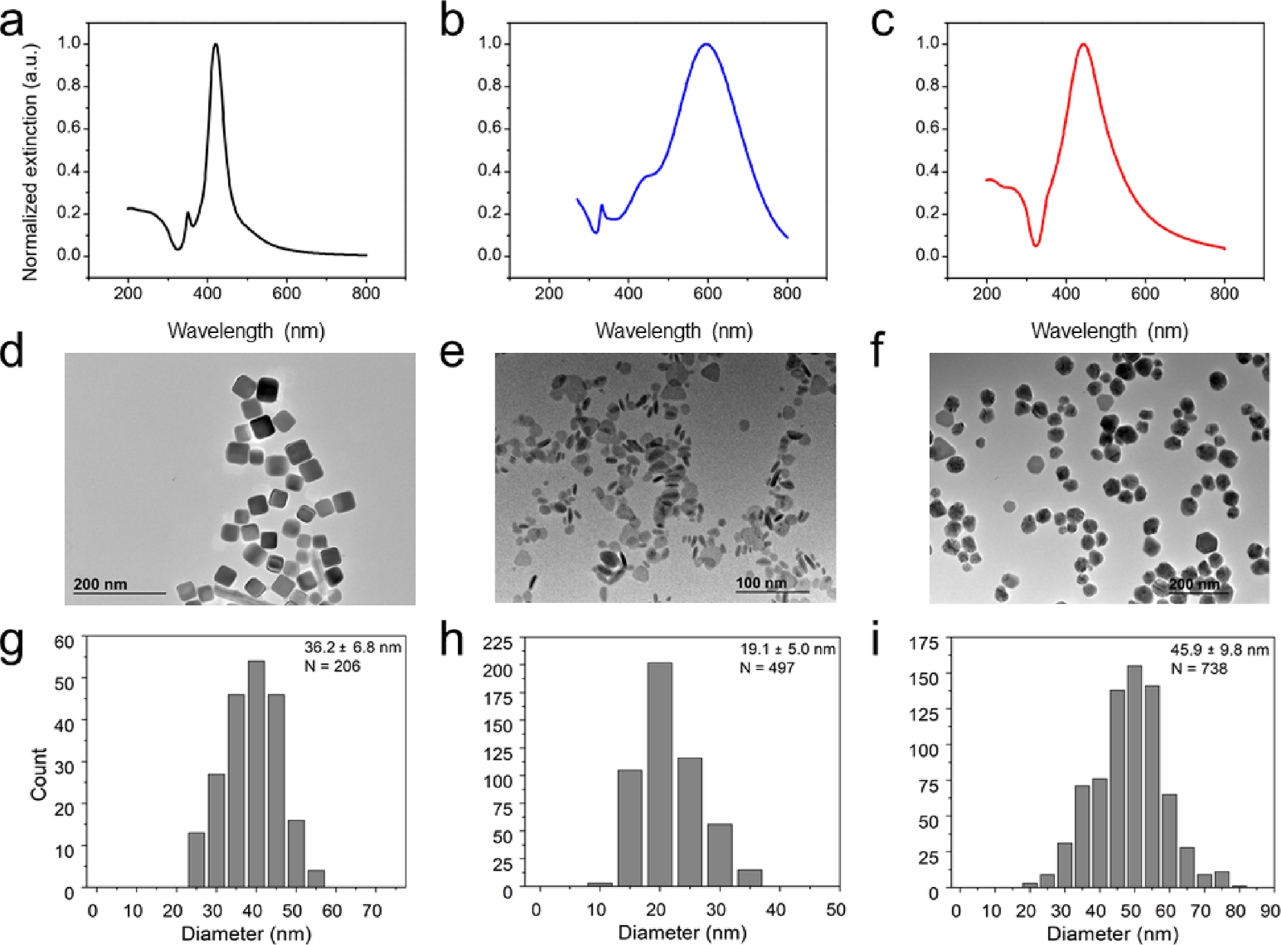
UV–vis–NIR and TEM characterization of AgNP shapes. (a–c) UV–vis–NIR absorption spectra show characteristic localized surface plasmon resonance (LSPR) peaks, (d,e) representative TEM images highlight the shape of the NPs, and (g–i) particle counts show the average NP size distribution for several batches of (a, d, g) cube-AgNPs, (b, e, h) disc-AgNPs, and (c, f, i) pseudospherical-AgNPs. Note that the TEM image of the disc-AgNPs reveal some discs lying flat and some on their sides, showing a thickness of 5.5 ± 1.3 nm. Some discs are truncated triangular in shape, while most are completely rounded.

PVP-capped pseudospherical-, cube-, and disc-AgNPs were synthesized according to previously reported procedures, which are detailed in the Supporting Information.^14,61,62^ PVP is a widely used, biocompatible, and slightly negatively charged ligand,^41,63^ which mitigates electrostatic attractive forces with negatively charged bacteria. All shapes exhibited a negative surface charge within a similar magnitude of each other (−31.10 ± 1.60 mV for disc-AgNPs, −25.91 ± 0.65 mV for cube-AgNPs, and −38.93 ± 1.45 mV for pseudospherical-AgNPs) and had similar measures of polydispersity and colloidal stability as determined by DLS/ELS (Table 1), thereby eliminating any substantial differences in surface charge, polydispersity, and stability among the AgNP shapes. In other words, all shapes were colloidally stable in ultrapure water (i.e., were electrosterically stabilized). After washing and purification, UV–vis–NIR spectra of the AgNPs revealed characteristic localized surface plasmon resonance (LSPR) peaks for each shape (Figure 1A–C). Single resonance peaks appear at λ_max_ ≈ 420 and 440 nm for the cube- and pseudospherical-AgNPs, respectively. For the discs, an in-plane dipole resonance peak appears at λ_max_ ≈ 600 nm, while the 450 and 350 nm peaks correspond with the out-of-plane dipole and quadrupole resonance peaks, respectively. The presence of these additional peaks indicates the formation of flat discs, and the blue shift of the in-plane dipole resonance to 600 nm (from a resonance peak at 770 nm for a perfect triangular nanoplate) suggests that the particles are truncated or rounded in shape. The average particle sizes of multiple independently synthesized batches were determined by TEM to be 36.2 ± 6.8, 19.1 ± 5.0, and 45.9 ± 9.8 nm for the cube-, disc-, and pseudospherical-AgNPs, respectively (Figure 1D–I, Table 1). Measurements of the hydrodynamic diameter (HDD) confirm the differences in particle size between the shapes, i.e., the cube- and pseudospherical-AgNPs are larger than the disc-AgNPs (Table 1), although it is important to note that the HDD is larger than the inorganic core size determined by TEM in all cases, which is expected given that the PVP ligand and the solvent layer surrounding the particle also contribute to the measured HDD. It is important to note that there can be significant batch-to-batch variability occurring with anisotropic AgNP shapes, particularly with disc-AgNPs because they can experience varying degrees of disc morphology (between disc-like and truncated triangular prisms) from etching, tip truncation, rounding, incomplete transformation, surface reorganization, and aging, which is common with silver nanoparticles.^11,13^ In the present study, some discs were truncated triangular, while most were found to be completely rounded, which corroborates the blue shift of the in-plane dipole resonance in the absorption spectra (Figure 1B) and explains the slightly higher polydispersity index for the disc-AgNPs compared to the other shapes (Table 1). Additionally, the TEM images of the disc-AgNPs reveal some discs lying flat and some on their sides, which enabled determination of the thickness (5.5 ± 1.3 nm, data not shown). Table 1 summarizes the geometries and surface areas of the AgNP shapes, and Table S1 provides a comparison of the shapes on an atom basis. Since the pseudospherical- and cube-AgNPs have comparable surface areas on a per particle basis, these two shapes enable a side-by-side comparison for shape-based antimicrobial activity outside the influence of surface area.

### Antimicrobial Activity of AgNP Shapes as a Function of Ag Mass Concentration

**Figure 2:**
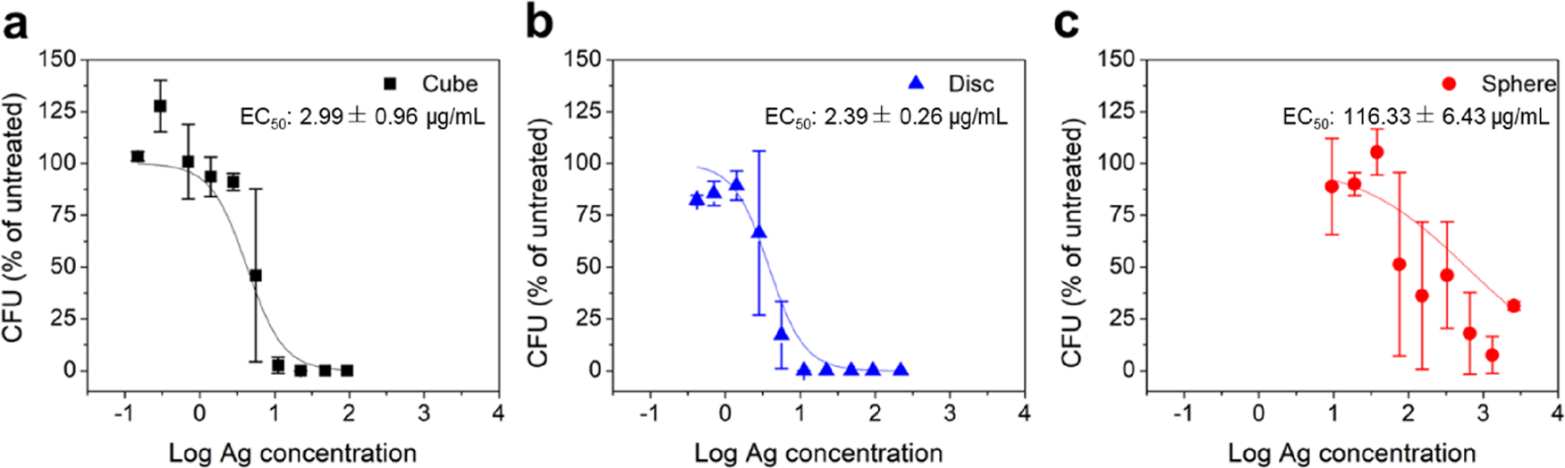
Antimicrobial activity of AgNPs toward *E. coli*. (a–c) Average sigmoidal dose–response curves after 3 h of contact time between AgNPs and *E. coli* using 0–200 μg/mL of the cube- and disc-AgNPs (and 0–1250 μg/mL for the pseudospherical-AgNPs). All experiments are compared to the negative control (no AgNPs) (*n* = 9) with (a) corresponding to cube-AgNPs, (b) disc-AgNPs, and (c) pseudospherical-AgNPs. The 95% confidence interval endpoints for the EC_50_ parameter can be found in Table 2.

**Figure 3:**
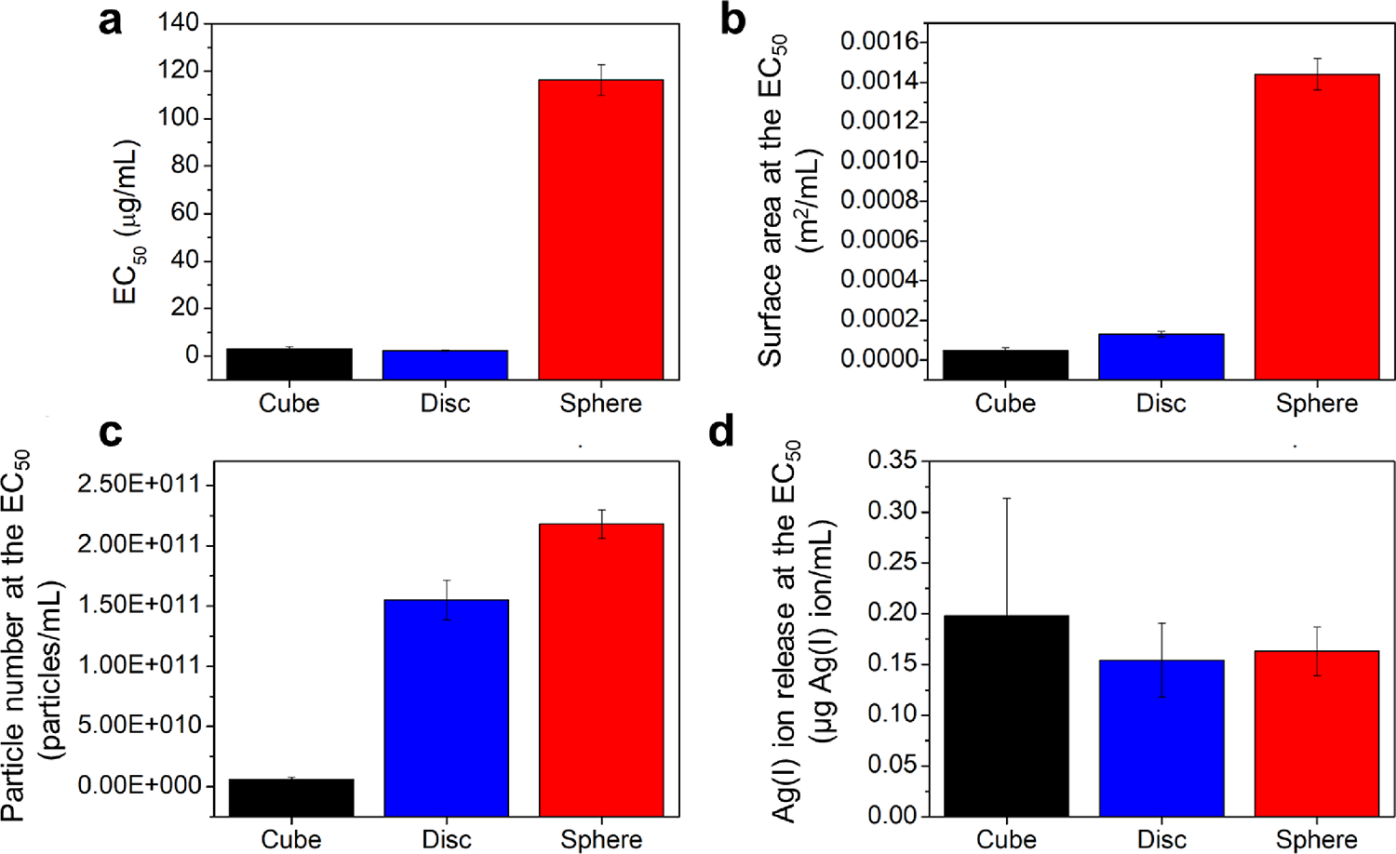
EC_50_ values of AgNP shapes for *E. coli* (3 h assay) as (a) mass-based concentration and converted to (b) surface area, (c) number of particles, and (d) ion release after 3 h in 0.9% saline. The 95% confidence interval endpoints for the EC_50_ parameter can be found in Table 2.

The antimicrobial activities of different AgNP shapes – cube, disc, and pseudosphere – were assessed by exposing *E. coli* to AgNP suspensions of concentrations ranging from 0 to 200 μg/mL (or 1250 μg/mL for the pseudospherical-AgNPs) for 3 h and enumerating CFU to generate dose–response curves. The EC_50_ was selected as the biological endpoint to compare antimicrobial activity across particle shapes and was determined from the sigmoidal dose–response curves (Figure 2) using a sigmoidal fit of (NB: three independent dose–response curves were generated from three biological replicates and the average values of three technical replicates at each particle concentration (*n* = 9)) (Figure S1). The EC_50_ was thus characterized by a reduction of bacterial cell viability determined by a decrease in CFU. Each nanoparticle shape reduced bacterial viability by 50% at different concentrations (Figure 3A). According to the dose–response curves, the calculated EC_50_ values were 2.99 ± 0.96, 2.39 ± 0.26, and 116.33 ± 6.43 μg/mL for cube-AgNPs, disc-AgNPs, and pseudospherical-AgNPs, respectively (Figures 2 and 3A, Table 2). The results indicate that the disc-AgNPs had the highest antimicrobial activity closely followed by the cube-AgNPs; however, the two shapes share overlapping 95% confidence intervals, suggesting that there is no statistically significant difference in their EC_50_ values and thus their antimicrobial activity (i.e., disc-AgNPs ⩾ cube-AgNPs). The pseudospherical-AgNPs exhibited the lowest antimicrobial activity however, and its 95% confidence interval did not overlap with the other two shapes, suggesting that it is statistically different from the other shapes (i.e., disc-AgNPs ⩾ cube-AgNPs ≫ pseudospherical-AgNPs).

### Antimicrobial Activity as a Function of Surface Area and Particle Number Reveals Different Trends across AgNP Shape

While we observe antimicrobial activity differences for different AgNP shapes, the underlying mechanism(s) through which shape imparts the observed difference remains to be determined. Herein, we suggest that differences in available surface area of differently shaped particles dosed at the same mass-based concentration may mask or lead to misinterpretation of comparative antimicrobial activity. In other words, the same mass of particles will introduce different surface area available for interactions with bacteria. Determining the role of surface area, if any, is therefore pursued here by converting the measured toxicity outcomes from mass to available surface area.

To estimate the per particle surface area for each shape, the average particle radius, height, and edge length were determined via TEM (assuming ideal sphere and cube geometries, and approximating discs as a cylinder) (Table 1). On a per particle basis, the cube-AgNPs and pseudospherical-AgNPs have comparable surface areas (Table 1, 7862.6 ± 2858.3 and 6615.4 ± 2830.2 nm^2^, respectively) while exhibiting substantially different antimicrobial activities (e.g., EC_50_ of 2.99 ± 0.96 and 116.33 ± 6.43 μg/mL, respectively). The disc-AgNPs exhibited the greatest antimicrobial activity (2.39 ± 0.26 μg/mL EC_50_ concentration) while having significantly lower surface area per particle (902.6 ± 386.9 nm^2^) (Table 1).

In addition to surface area, the total number of atoms and surface atoms are important to consider because differences at the atomic level (i.e., atom density, atom arrangement) can impart unique surface energies and reactivities (Table S1). The disc-AgNPs have significantly less total and surface atoms per particle (Table S1), which is expected given the particle anisotropy and a high surface area-to-volume ratio (0.57 ± 0.06 nm^–1^) compared to the cube-AgNPs (0.17 ± 0.03 nm^–1^) and the pseudospherical-AgNPs (0.13 ± 0.03 nm^–1^). These values indicate that a larger proportion of atoms can be found on the surface of the disc-AgNPs (13% versus 2.8% and 3.38% for the pseudospherical and cube-AgNPs, respectively), which may suggest the importance of atom arrangement (i.e., the proportion of atoms positioned at the surface) compared to per particle surface area alone.

Differently shaped AgNPs dosed at the same concentrations will present different total available surface area in the experimental system. Since interactions that lead to cell inactivation begin at the particle surface, difference in total surface area will influence the measured antimicrobial outcome. The theoretical total available surface area in each AgNP shape system (assuming no aggregation) at the EC_50_ (and at the equivalent mass-based concentrations in the dose–response curves from Figure 2) was calculated as an alternative to the mass-based concentration. This enables determination of the surface area needed to induce the same magnitude of response (i.e., 50% inactivation of the bacteria population) (note: each equivalent mass-based concentration across the three AgNP shapes yielded different total surface areas). The per particle surface area (*vide supra*), estimated number of atoms, and estimated number of particles at the different AgNP EC_50_ concentrations were calculated to provide insight into surface area and particle number influences on the observed differences in AgNP antimicrobial activity (Table 2, see Table 1 and Table S1 for the per particle surface area and conversion factors used in these calculations). The measured outcome, in this case the EC_50_, can then be converted to surface area and particle number (Figure 3B,C) to see how these factors contribute to the differences observed in shape-based antimicrobial activity (Figure 3A).

Comparing the pseudospherical- and cube-AgNPs suggests that there may not be a direct correlation between surface area and antimicrobial activity (i.e., greater surface area does not result in greater antimicrobial activity) and/or that surface area alone does not influence antimicrobial activity. The cube-AgNPs have significantly less total available surface area (Table 2, 4.70 × 10^–5^ ± 1.51 × 10^–5^ m^2^/mL) and the lowest particle concentration (5.97 × 10^9^ ± 1.92 × 10^9^ particles/mL) at the EC_50_ (Table 2), whereas the pseudospherical-AgNPs have significantly higher total surface area (1.44 × 10^–3^ ± 7.94 × 10^–5^ m^2^/mL) and the highest particle concentration (2.18 × 10^11^ ± 1.17 × 10^10^ particles/mL) at the EC_50_ (Table 2). When the EC_50_ concentrations are converted to both surface area and particle number (Figure 3B,C and Table 2), the relative trend in antimicrobial activity changes (i.e., cube-AgNPs > disc-AgNPs > pseudospherical-AgNPs and cube-AgNPs ≫ disc-AgNPs ⩾ pseudospherical-AgNPs, respectively). The differences in antimicrobial activity between the cube-AgNPs and disc-AgNPs become more pronounced (i.e., cube-AgNPs ≫ disc-AgNPs), and they no longer share overlapping 95% confidence intervals, suggesting that they are statistically different. The difference in antimicrobial activity between the disc-AgNPs and pseudospherical-AgNPs decreases (i.e., disc-AgNPs ⩾ pseudospherical-AgNPs), although there is no overlap in their 95% confidence intervals, suggesting that their antimicrobial activities are still statistically different. In other words, converting to surface area and particle number eliminates the approximate 50× difference in the potency of the disc- and pseudospherical-AgNPs observed on a mass basis such that the disc-AgNPs are only 10× as potent as pseudospherical-AgNPs based on surface area (Table 2, Figure 3B) and 1.5× as potent based on particle number (Table 2, Figure 3C). The cube-AgNPs remain the most potent particle shape (EC_50_-surface area: 4.70 × 10^–5^ ± 1.51 × 10^–5^ m^2^/mL, EC_50_-particle: 5.97 × 10^9^ ± 1.92 × 10^9^ particles/mL) (Table 2). Converting the mass-based EC_50_ to surface area differentiates the shapes, with cube-AgNPs 3× as potent as the disc-AgNPs. Considering antimicrobial activity based on particle number further differentiates these shapes with an order of magnitude increase in potency (i.e., cube-AgNPs are 30× more potent than disc-AgNPs).

This statistically significant shift in relative antimicrobial activity for the disc-AgNPs, upon converting to available surface area in the system, preliminarily suggests that the difference in available surface area in relation to the pseudospherical-AgNPs partly drives the difference in observed mass-based concentration antimicrobial activity (Figures 1 and 3A). The comparatively high antimicrobial activity of the cube-AgNPs, when converted to surface area, suggests that surface area is likely not driving the observed behavior for this shape and there is likely an additional factor(s) underlying its antimicrobial activity. Further, AgNP size is not driving the behavior (i.e., the smaller disc-AgNPs are not more active) (Table 1). Based on these results alone, surface area likely plays a role in, but is not a universal property, driving the antimicrobial activity of different AgNP shapes.

**Figure 4:**
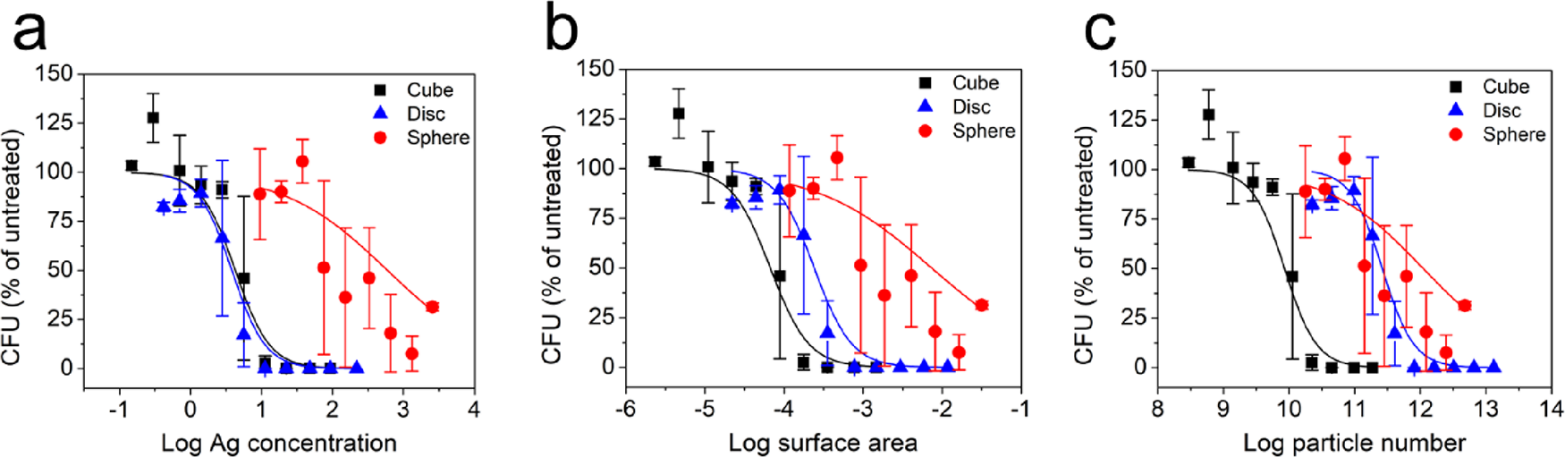
Average sigmoidal dose–response curves in units of (a) Ag concentration (μg/mL) (data reprinted from Figure 2), (b) surface area (m^2^/mL), and (c) particle number (particles/mL). The 95% confidence interval endpoints for the EC_50_ parameter can be found in Table 2.

To further investigate the influence of shape and exposure system conditions on the observed antimicrobial activity, we considered trends of the entire dose–response curve (Figure 2) rather than the single, EC_50_ data point. The shape and location (i.e., shifts left and right) of these curves as a function of mass-, surface area-, and particle-based concentrations can illuminate the influence of these factors on interactions with bacteria that result in cell inactivation. In addition, the position of the curves for the different shapes suggests relative toxicity within a given dose metric. When the horizontal axis of the sigmoidal dose–response curves is converted from Ag concentration to the associated equivalent surface area and number of particles (i.e., transposed using the total m^2^ per concentration dose based on differing surface area for each shape) (Figure 4), there is a notable shift to the right for the disc-AgNPs relative to the other shapes. A right shift in the relative location of the dose–response curve indicates that significantly more of the unit dose metric (more mass, more surface area, more particles) is required to elicit the same bacterial response (here, bacteria inactivation). The disc-AgNPs curve overlaps that of cube-AgNPs on a mass-based dose metric, overlaps pseudospherical AgNPs on a particle-based dose metric, and falls between these two shapes on a surface area-based dose metric. The position of these curves indicates the relative amount of each shape needed to induce the equivalent response, and the position shift for the disc-AgNPs suggests that this shape is most sensitive to the dose metric. This could be due to their low surface area, volume, and atom number on a per particle basis as well as their anisotropy such that they require significantly higher amounts of surface area and particles to compensate for. Converting to either property of the disc particle system as a dose metric decreases their antimicrobial activity relative to the cubes. It also suggests that the available surface area and number of particles present influences the bacterial response to disc-AgNPs and are factors underlying their antimicrobial activity. Since the relative location of the dose–response curve for the cube-AgNPs remains the same (i.e., the cubes consistently require low concentration, surface area, and particle number to induce the same response), surface area and particle number may play a role in the observed behavior, but there is likely another property that supersedes these, governing the antimicrobial activity of the cube-AgNPs.

### Empirical Investigation of the Importance of Surface Area on Antimicrobial Activity

To empirically probe the proposed relationship between shape, surface area, and antimicrobial activity, bacterial inactivation was evaluated for each shape dosed at equivalent total available surface area. Further, the available surface areas of each shape at their respective EC_50_ concentrations are differentiated by orders of magnitude, which could confound the theoretical exercise presented above. Considering the above theoretical results alongside this empirical study intends to bring clarity to the influence of particle shape on antimicrobial activity mechanisms.

**Figure 5:**
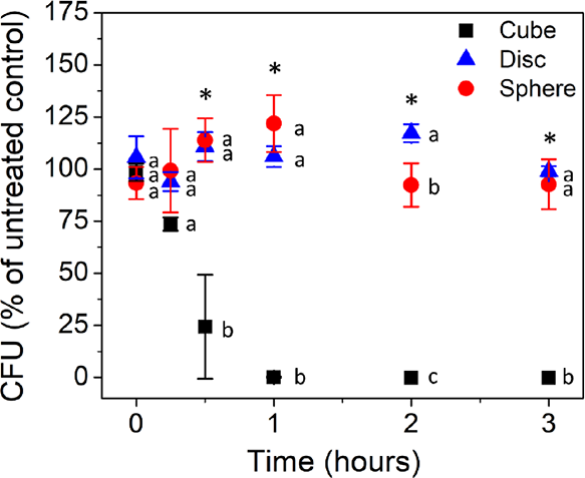
*E. coli* response after 3 h to AgNP shapes dosed in at particle concentrations having an equal available surface area (i.e., 1.40 × 10^–4^ m^2^mL^–1^): 2.6, 9.0, and 11.4 μg/mL for disc-, cube-, and pseudospherical-AgNPs, respectively. The data were normalized to the control (0.9% NaCl without AgNPs), and the cell concentration of the control remained constant after the 3 h incubation. Means suffixed with different letters (a–c) for each time point are significantly different from each other at *p* < 0.05. Error bars denote the standard deviations of sample replicates.

Inactivation experiments were carried out at an equivalent total available surface area of 1.40 × 10^–4^ m^2^mL^–1^, which translates into 2.6 μg/mL (at the EC_50_) for disc-AgNPs, 9.0 μg/mL (above the EC_50_) for cube-AgNPs, and 11.4 μg/mL (below the EC_50_) for pseudospherical-AgNPs, confirmed by UV–vis–NIR and ICP-MS/OES. The results (Figure 5) demonstrate that different antimicrobial activities are maintained across the AgNP shapes (i.e., 100% inactivation for cube-AgNPs, <25% inactivation for disc- and pseudospherical-AgNPs), with the same relative trend (i.e., cube-AgNPs ≫ disc-AgNPs ⩾ pseudospherical-AgNPs) as obtained when converting the sigmoidal dose–response curves to different dose metrics by calculation (*vide supra*, Figure 4). Notably, when considering equivalent surface area in the different shape systems, the potencies of the disc-AgNPs and pseudospherical-AgNPs are not statistically different, which suggests that the difference in available surface area in relation to the pseudospherical-AgNPs partly drives the difference in the initial observed mass-based concentration antimicrobial activity that is eliminated when surface area is held constant. Additionally, the fact that the cubes are increasing in potency in relation to the disc- and pseudospherical-AgNPs, even as surface area is held constant, suggests that factors other than surface area are governing their antimicrobial activity. Still, we investigate one equivalent surface area in this experiment and acknowledge that selection of additional equivalent surface areas along the dose–response curve (Figure 4B) could elicit different relative trends in antimicrobial activity. For example, at high equivalent surface areas along the dose–response curve, the relative trends would likely shift to disc-AgNPs ≈ cube-AgNPs (no differential response is observed) > pseudospherical-AgNPs or to disc-AgNPs ≈ cube-AgNPs ≈ pseudospherical-AgNPs at low equivalent surface areas (Figure 4B).

Most studies investigating shape-dependent antimicrobial activity include either a single or a small range of mass-based equivalent doses, which may contribute to the varied and inconclusive reported findings. The location along the dose–response curve that the mass-based concentrations are selected can contribute to conclusions that do not distinguish across shapes (i.e., the selected mass-based AgNP concentration and associated surface areas and particle numbers may not impart differential bacterial response across the studied shapes). This can also contribute to the conclusions that draw specific trends in shape-based antimicrobial activity (i.e., the selected mass-based AgNP concentrations can occur at points along the dose–response curve that result in distinct responses and/or lead to significantly different total surface areas across the studied shapes). Since total surface area is not quantified nor considered across different shapes, its influence on antimicrobial activity has not been comprehensively considered until now. Our results underline the importance of selecting particle concentrations and including a range of dose metrics (e.g., surface area-, particle number-, and mass-based concentrations) within a study aimed at uncovering shape effects. Studying a range of equivalent total surface areas will uncover relationships between the antimicrobial activity mechanism(s) of interest and surface area as well as whether those relationships are preserved across a large surface area range.

### Surface Facet Influence on Cube-AgNP Antimicrobial Activity

In addition to the total available surface area, shape introduces different crystallographic structure, which gives rise to varying crystal facets at the particle surface^11,13,14,18,26^ and enables distinct physical interactions with the bacteria cell. Crystal facets form when the surfaces along specific directional planes grow at different rates (also imparted by the adsorption of capping ligands to a specific crystallographic surface) and are denoted by Miller indices^33^ that indicate the coordinates of those planes.^11,13,14^ The three low index basal planes,^9−11,13,30^ {100}, {110}, and {111} are especially important in determining a crystal’s geometric shape. Disc-AgNPs are rich in {111} facets (with varying percentages of {100} facets depending on their thickness and degree of truncation),^9−11,13,17,18,25,30,32,39^ while cube-AgNPs are unique in that they are rich in {100} facets. The pseudospherical-AgNPs are approximated as cuboctahedra having eight {111} faces and six {100} faces and thus contain a mix of {100} and {111} facets (Table 1).^26,33^ These facets impart different surface energies, atom densities, arrangement of atoms on the surface, bonding, presence of defect sites, and electronic structure, leading to differential facet reactivity.^13,26−28^ Both cube- and disc-AgNPs have heterogeneous surfaces, including corners and edges, that are suggested to be more biologically and chemically reactive, arising from the associated atoms having a lower bonding coordination (i.e., weaker bonds) than bulk atoms.^27^ Thus, crystal facets and the presence of heterogeneous surfaces should be considered factors that are a consequence of particle shape and may drive antimicrobial activity. Further, the facets of the cube-AgNPs may underlie their unique observed activity.

Enhanced activity and shape effects induced by the increased reactivity of certain crystal facets is identified in studies of different AgNP shapes.^26,33^ For example, the higher surface energy {100} facets on cube-AgNPs are more reactive compared to spheres that are covered with relatively stable, lower surface energy {111} facets.^33,64−67^ This is contrary, however, to studies that describe higher reactivity and antimicrobial activity of the {111} facets,^17,18,22,25,30,32,39^ introducing a common point of debate because the surface energies of the low index basal planes for noble metals generally follow the order of γ{111} < γ{100} < γ{110}.^13,26,68^

Of the few studies that compare the same shapes: cubes-pseudospheres-discs (with ‘discs’ also referring to ‘plates’, ‘prisms’, or ‘triangles’ depending on the degree of truncation and rounding^13,14^), the common conclusion is that cube-AgNPs exhibit significantly lower antimicrobial activity.^23−25^ These studies commonly attribute the greater antimicrobial activity of disc- and pseudospherical-AgNPs to their higher surface areas and possession of high atom density {111} facets allowing for more dissolution (as monitored by ICP or AAS) than cube-AgNPs possessing less available surface area. However, critical limitations of these studies include exclusion of surface area dose metrics and/or quantification of nanoparticle surface area. The cube-AgNPs synthesized in this study have significantly less total available surface area at the EC_50_ compared to the other shapes and yet exhibit significantly higher antimicrobial activity, suggesting that the presence of high-energy {100} crystal facets may be the main driver of the high particle-specific antimicrobial activity observed in this study. Given that differential antimicrobial activity was observed for the AgNP-shapes having equal surface area in our study, it is possible that each shape may impart different effective surface areas in terms of active facets. Cube-AgNPs likely have a greater effective surface area in terms of active {100} facets,^26^ while the anisotropy of the disc-AgNPs may limit their effective surface area by reducing the amount of active {100} facets. Disc-AgNPs are 2D, flat-lying cylinders with relatively stable {111} facets on the top and bottom basal planes and {100} facets on their thin edges.^9−11,13,18^ Anisotropic growth and etching mainly occur from the side, effectively reducing the amount of active {100} facets in their final shape and thus limiting their availability for interaction with bacteria cells.^11,13,14^ Thus, it may be that possession of {100} crystal facets strongly impart the antimicrobial activity of cube-AgNPs, whereas surface area and particle number likely dominate the antimicrobial activity of disc-AgNPs and supersede the influence of the {111} facets. This facet-dependent reactivity in the case of cube-AgNPs can influence enhanced binding affinity with the bacteria cell membrane and interaction with the oxygen-containing groups of the lipopolysaccharide molecules to induce cell membrane damage as well as other downstream physical and chemical inactivation mechanisms.^33^

### Antimicrobial Activity of AgNP Shapes as a Function of Ag(I) Ion Release

Lastly, since the release of Ag(I) ions is a well-documented factor contributing to AgNP antimicrobial activity, we monitored ion release (measured as total Ag in the supernatant) for all particle shapes at their respective EC_50_ concentration. The influence of Ag(I) ion release on shape-based antimicrobial activity is discussed in the Supporting Information (Figure 3D, Figures S2 and S3). Based on the data collected here, we cannot say if the Ag(I) ions alone are driving the differences in antimicrobial activity across shape nor can we rule out the presence of other particle-specific factors contributing to the differences in antimicrobial activity. Thus, we cannot draw any conclusions surrounding the influence of Ag(I) ions on the differences in antimicrobial activity across shape. A more robust set of experiments evaluating Ag(I) ion release is thus needed to clarify and unravel these competing mechanisms of shape-based antimicrobial activity.

## Conclusions

This work suggests that particle shape is a viable design handle for manipulating properties associated with AgNP antimicrobial activity. Although AgNPs have been widely studied and employed in numerous antimicrobial applications, only a few studies explore shape-based antimicrobial activity. Herein, we considered dose metrics other than mass-based concentration to elucidate complexities in unraveling particle-specific mechanisms influencing different shape-based antimicrobial potency. We carefully controlled particle syntheses to establish three particle shapes of controlled physical and chemical properties that enabled comparisons of antimicrobial activity based on equivalent surface area. Our collective results suggest that evaluation of particle shape on ionizing ENM antimicrobial activity must be considered with factors such as available surface area and particle number as biologically relevant dose metrics to reveal drivers of antimicrobial activity. In our study, the antimicrobial potency of the disc-AgNPs was determined to be sensitive to the dose metric, significantly decreasing in potency (∼5–30×) upon conversion from a mass-based concentration to surface area and particle number, whereas the cube-AgNPs maintained their high potency and the pseudospherical-AgNPs their low potency no matter the dose metric being compared. Specifically, when using a mass-based dose metric, the disc-AgNPs presented the highest antimicrobial activity of the three shapes (EC_50_: 2.39 ± 0.26 μg/mL for disc-AgNPs, 2.99 ± 0.96 μg/mL for cube-AgNPs, 116.33 ± 6.43 μg/mL for pseudospherical-AgNPs). When surface area and particle number were used as dose metrics, the disc-AgNPs decreased in their antimicrobial potency such that the cube-AgNPs possessed the highest antimicrobial activity (EC_50_-surface area: 4.70 × 10^–5^ ± 1.51 × 10^–5^ m^2^/mL, EC_50_-particle: 5.97 × 10^9^ ± 1.92 × 10^9^ particles/mL), signifying that the antimicrobial activity of the different shapes are governed by different particle-specific factors. Surface area likely plays a role in, but is not a universal property, driving the differences in antimicrobial activity between all the shapes. To further support this, at equivalent particle surface areas, significantly different magnitudes of antimicrobial activity were observed for the different shapes (i.e., 100% inactivation for cube-AgNPs, <25% inactivation for disc- and pseudospherical-AgNPs). Thus, differences in surface area help explain the difference between the potency of the disc- and pseudospherical-AgNPs, but other particle variables such as crystal facets are likely superseding the influence of surface area and particle number on the cube-AgNPs that remain high in potency throughout the study. Taken together, these results signify that the cube-AgNPs likely participate in other types of antimicrobial activity (e.g., facet-dependent) while disc-AgNPs may participate namely in surface area-dependent antimicrobial activity. Future research investigating different AgNP shape effects on Gram-positive organisms would elucidate the sensitivity of particle shape effects (surface area, crystal facet) to cell wall architecture (i.e., the lack of an outer membrane and presence of a thick peptidoglycan layer). In addition to traditional mass-based dose metrics, we demonstrate, and champion, surface area and particle number as biologically relevant dose metrics for evaluation of ENM antimicrobial activity. Revealing shape-dependent, particle-specific properties of AgNPs, and other ENMs, on microbial interactions is critical to leveraging shape control for manipulating antimicrobial activity relevant to nanotoxicity and antimicrobial applications.

## Supporting Information Available

Synthesis methods for pseudospherical-, cube, and disc-AgNPs; methods for Ag(I) ion release quantification; normalization calculations; CFU counts; Ag(I) ion release profiles; and TEM particle degradation (PDF)
