# Supplementary material for: Particle-Driven Effects at the Bacteria Interface: A Nanosilver Investigation of Particle Shape and Dose Metric
Source: ACS Appl Mater Interfaces. 2023 Aug 15;15(33):39027–38. doi: 10.1021/acsami.3c00144 (PMC10450641; doi:10.1021/acsami.3c00144)
Supplement: Supplementary file 1 — am3c00144_si_001.pdf [file am3c00144_si_001.pdf]

Supporting Information

Particle-driven effects at the bacteria interface: A nano-silver investigation of particle shape and dose metric

Lisa M. Stabryla,[a*] Paige J. Moncure,[b] Jill E. Millstone,[b,c,d] Leanne M. Gilbertson[a,c*]

[a]Department of Civil and Environmental Engineering, University of Pittsburgh, 3700 O'Hara Street, Pittsburgh, PA 15261

[b]Department of Chemistry, University of Pittsburgh, 219 Parkman Avenue, Pittsburgh, PA 15260

[c]Department of Chemical and Petroleum Engineering, University of Pittsburgh, 3700 O'Hara Street, Pittsburgh, PA 15261

[d]Department of Mechanical Engineering and Materials Science, University of Pittsburgh, 3700 O'Hara Street, Pittsburgh, PA 15261

Table of Contents

Synthesis and ligand exchange to PVP-capped pseudospherical-AgNPs……………...…P.S-2

Synthesis of PVP-capped cube-AgNPs……..……….……………….…..………….………...P.S-3

Synthesis of PVP-capped disc-AgNPs…………………………………………………….....P.S-4

Monitoring Ag(I) ion release………………………………………………………………...P.S-5

Example normalization calculations…….…………….……………….……….……….…..P.S-6

Table S1. Comparison of shape on an atom basis……………………………….………....P.S-7

Figure S1. Viable CFU count by shape……………………………………………………P.S-7

Antimicrobial activity of AgNP shapes as a function of Ag(I) ion release……………….P.S-8

Figure S2. Ag(I) ion release profiles by shape………………...…………………………..P.S-10

Figure S3. Particle degradation by TEM.……………………...…….………………………P.S-10

References…………………………………………………………..……………………P.S-11

*co-corresponding authors: lms162@pitt.edu; leanne.gilbertson@pitt.edu

Synthesis and ligand exchange to PVP-capped pseudospherical-AgNPs

Citrate-capped pseudospherical-AgNPs (diameter, *d* = 27.2 ± 4.8 nm) were synthesized and functionalized with PVP (average MW = 55,000 Da) according to a previously reported procedure [1]. It is important to note that particles having a diameter of *d* = 45 nm were targeted using increased concentrations of tannic acid and silver nitrate. Briefly, a 100 mL aqueous solution of citrate (5 mM) and tannic acid (5 mM) was brought to reflux in a 250 mL, three-neck round-bottom flask while stirring at a rate of 340 rpm. The molar ratio of tannic acid to citrate was 1:1 to achieve ~ 45 nm AgNPs [1]. Once 100 °C was reached and a rapid drip rate was achieved (drip rate ~one/s), 1.00 mL of $AgNO_3$ (330 mM) was quickly injected. The reaction mixture changed from a transparent, pale-yellow color (due to the tannic acid) to a dark, golden brown within one minute. The resulting AgNP solution was allowed to reflux for 5 min before it was removed from heat and cooled to room temperature by sitting on the benchtop. The citrate-capped AgNPs were concentrated and washed to remove excess citrate and tannic acid by centrifuging 1.50 mL aliquots in 1.50 mL centrifuge tubes at ~20,000 rcf for 6 min (Eppendorf 5424 centrifuge). The supernatants were removed and discarded, and the pellet was re-suspended in another 1.50 mL aliquot of AgNP solution from the round bottom flask and centrifuged again. After the second removal of the supernatants, the particles were re-suspended in 1.00 mL of H_2O. The prepared citrate-capped AgNPs, as 1.00 mL H_2O suspensions, were functionalized with PVP (average MW = 55,000 Da) using mass action ligand exchange (Scheme S1 in Ref [2]). Here, 5 mM PVP was added to each tube containing the particles in 1.00 mL H_2O. These mixtures were vortexed and then placed on a temperature-controlled mixer (Eppendorf R Thermomixer) in the dark at 600 rpm and 25 °C for 24 h. Immediately after mixing, the PVP-capped AgNPs were further concentrated and purified with a series of washing steps to remove excess PVP and displaced citrate. Here, 1.00 mL aliquots

were centrifuged, and their supernatants were removed. Two of the pellets were combined with 1.00 mL of H_2O so that half of the number of total tubes remained. This process of combining pellets after centrifuging was repeated several times, keeping the same 1.00 mL total volume, until one concentrated tube remained. The particles were then washed by centrifuging once more, the supernatant was removed, and the particles were re-suspended in 1.00 mL of H_2O to provide sufficient volume of concentrated AgNPs for the experiments. Multiple 10.0 µL aliquots of this solution were then diluted in 1.00 mL of H_2O to (i) obtain a concentration that would fall under the upper limit of quantification to reliably measure the initial AgNP stock concentration by ultraviolet-visible-near infrared (UV-vis-NIR) spectroscopy, (ii) provide good resolution for characterizing particle size by transmission electron microscopy (TEM), (iii) fall within the specifications for inductively coupled plasma mass spectrometry (ICP-MS) and optimal emission spectrometry (ICP-OES) to confirm the AgNP stock concentration obtained with UV-vis-NIR, and (iv) enable analysis of surface charge and particle stability with dynamic light scattering (DLS) and electrophoretic light scattering (ELS). To determine the total Ag atom content, the absorption spectrum of the solution was measured to locate the λ_{max}. Then, using a calibration curve (Figure S3 in Ref [2]) that plots extinction at λ_{max} vs. the concentration of Ag atoms as measured by ICP-MS, the concentration of Ag in the original stock solution was determined. The remaining solution was used in the antimicrobial activity assays (**Figure S1**).

Synthesis of PVP-capped cube-AgNPs

PVP-capped cube-AgNPs were synthesized according to a previously reported procedure [3]. Briefly, 6.00 mL of ethylene glycol (EG) was heated in an oil bath to 155°C for one hour with stirring at 300 rpm, with care to maintain the temperature at 155°C to obtain high quality, monodispersed cubes as the etching process is very temperature-sensitive and can lead to the

presence of other shapes. After one hour, a trace amount of sodium hydrosulfide (80 µL, 3mM in EG) was injected, followed by PVP (average MW = 55,000 Da, 1.5 mL, 20 mg/mL in EG) and $AgNO_3$ (0.5 mL, 48 mg/mL in EG). The molar ratio of PVP to $AgNO_3$ was 1.9:1, which is also important in producing uniform cubes since PVP binds to 100 surfaces and directs the final faceted shape (Siekkinen et al. 2006). The solution was left to react for roughly eight minutes, during which the color changed from clear to transparent yellow orange almost instantaneously, followed by additional changes to an opaque, opalescent ruddy green or whitish-brown. The cube-AgNPs were then purified in 0.5 mL aliquots by adding 0.5 mL acetone and centrifuging at ~20,000 rcf for 10 min to remove excess PVP and EG. The supernatants were discarded, and the pellet was re-suspended in 1.00 mL of H_2O and centrifuged again. After the second removal of the supernatants, the particles were re-suspended in 1.00 mL of H_2O. A series of slow-spin washes (i.e., 3-4) proceeded, where the particles were centrifuged at 5,000 rpm for 1.5 min. Here, the supernatants were kept and transferred to a new tube. This was done to remove any larger, irregular-shaped particles (i.e., wires) that were present and pelleted at the bottom. The cube-AgNPs were then further concentrated with the same series of washing and combining steps mentioned above for the pseudospherical-AgNPs until one concentrated tube remained. The particles were again re-suspended in 1.00 mL of H_2O for sample characterization by UV-vis-NIR, TEM, ICP-MS/OES, and DLS/ELS, and for use in the antimicrobial activity assays (**Figure S1**).

Synthesis of PVP-capped disc-AgNPs

PVP-capped disc-AgNPs were synthesized according to a previously reported procedure [4]. Briefly, a 24.75 mL aqueous solution containing $AgNO_3$ (0.05 M, 50 µL), trisodium citrate (75 mM, 0.5 mL), PVP (MW = 29,000 Da, 17.5 mM, 100 µL) and H_2O_2 (30 wt %, 60 µL) was

vigorously stirred at room temperature. Sodium borohydride (100 mM, 250 μL) was then rapidly injected. Within one hour, the clear solution transitioned into a deep yellow, followed by changes to a magenta and finally a deep royal blue or purple color. The disc-AgNPs were then purified in 1.00 mL aliquots by centrifuging at ~20,000 rcf for 6 min. The supernatants were kept, transferred to a new tube, and subjected to the same series of washing and concentrating steps mentioned above for the other shapes until one concentrated tube remained. The particles were again re-suspended in 1.00 mL of H_2O for sample characterization by UV-vis-NIR, TEM, ICP-MS/OES, and DLS/ELS, and for use in the antimicrobial activity assays (**Figure S1**).

Monitoring Ag(I) ion release

Similar to surface area, Ag(I) ion release is another confounding factor in the system and so its influence on shape-based antimicrobial activity (**Figure S1**) was explored by monitoring the amount of Ag(I) ions in each shape's system over three hours using ICP-MS and ICP-OES. Ag(I) ion release in 0.9% NaCl (n=2) was monitored at particle concentrations selected based on each shape's EC_{50} concentration to ensure that any differences in Ag(I) ion release could be attributed to the same level of inactivation as well as meet the instrument's limit of detection. AgNPs (50 μL) were added to 50 μL 0.9% NaCl in 96 well plates to obtain each shape's EC_{50} concentration. The plate was incubated at 37 °C for 3 h with medium linear shaking (~500 cpm) to mimic the inactivation experiments. Twelve wells were pooled together to comprise one sample for each time point over three hours (t = 0, 0.25, 0.5, 1, 2, and 3 h) and centrifuged at 17,000 rcf for 15 minutes. The supernatant was obtained (~750-1,000 μL), being careful not to disturb the pellet. It is important to note that for the anisotropic shapes (i.e., the cube- and disc-AgNPs), the pellet would collect on the side rather than the bottom of the tube and so extra care had to be taken to withdraw the supernatant. The disc-AgNPs were also subjected to an additional centrifuge spin (30 minutes

total) to obtain a pellet. Following this, a 5% (by volume) nitric acid matrix solution was prepared with nitric acid and diluted with NANOpure water. A few drops of concentrated, ultrapure nitric acid (~125 µL) were then added to the obtained supernatants, which were further diluted to 2 mL using the 5% nitric acid matrix (note: 10 mL of the 5% nitric acid matrix was used in the case of the more concentrated AgNP stock solutions, typically starting with volumes of 800-950 µL). Total Ag was measured with ICP-MS and ICP-OES (limit of detection between 1-10 ppb). In the case of ICP-MS, the concentrated stock samples were diluted by an additional factor of 16 (0.25 mL in 3.75 mL 5% nitric acid) to fit within the upper limit of quantification of the instrument. The ICP-MS and ICP-OES analysis was performed using an argon flow with a NexION spectrometer (PerkinElmer, Inc.) (Figure S3 in Ref [2]). Unknown Ag concentrations were determined by comparison to a 7-point standard curve with a range of 1 - 100 ppb (ICP-MS) or 0.1-10 ppm (ICP-OES) from a silver standard for ICP (Fluka, TraceCERT 1,001 ± 2 mg/L Ag in HNO_3) diluted in the 5% nitric acid matrix. All standards were measured 5 times and averaged, while all unknown samples were measured in triplicate and averaged. A 5-minute flush time with 5% nitric acid matrix was used between all runs, and a blank was analyzed before each unknown sample to confirm removal of all residual metals from the instrument.

Example normalization calculation (corresponding to cube-AgNPs)

Mass-based EC_{50} (µg/mL) to surface-normalized EC_{50} (m^2/mL)

$$2.99\ \frac{\mu g}{mL} * \frac{1 g}{10^6\ \mu g} * \frac{1\ mol}{107.87\ g} * \frac{6.02\ E\ 23\ atoms}{1\ mol} * \frac{1\ particle}{2{,}793{,}864\ atoms} * 7862.6\frac{nm^2}{particle} * \left(\frac{1\ m}{10^9\ nm}\right)^2 = 4.70E^{-5}\ \frac{m^2}{mL}$$

Mass-based EC_{50} (µg/mL) to particle-normalized EC_{50} (particles/mL)

$$2.99\ \frac{\mu g}{mL} * \frac{1g}{10^6\ \mu g} * \frac{1\ mol}{107.87\ g} * \frac{6.02\ E\ 23\ atoms}{1\ mol} * \frac{1\ particle}{2{,}793{,}864\ atoms} = 5.97E^9\ \frac{particles}{mL}$$

Mass-based EC_{50} (μg/mL) to Ag atom-normalized EC_{50} (m^2/mL)

$$2.99\ \frac{\mu g}{mL} * \frac{1g}{10^6\ \mu g} * \frac{1\ mol}{107.87\ g} * \frac{6.02\ E\ 23\ atoms}{1\ mol} = 1.67E^{16}\ \frac{Ag\ atoms}{mL}$$

Mass-based EC_{50} (μg/mL) to Ag surface atom-normalized EC_{50} (m^2/mL)

$$2.99\ \frac{\mu g}{mL} * \frac{1g}{10^6\ \mu g} * \frac{1\ mol}{107.87\ g} * \frac{6.02\ E\ 23\ atoms}{1\ mol} * \frac{1\ particle}{2{,}793{,}864\ atoms} * \frac{94{,}466\ Ag\ surface\ atoms}{1\ particle} = 5.60E^{14}\ \frac{Ag\ surface\ atoms}{mL}$$

Table S1. Comparison of shape on an atom basis.

	Cubes	Discs	Pseudospheres
Estimated total number of atoms per particle	2,793,864 atoms	92,811 atoms	2,982,059 atoms
Estimated total number of surface atoms per particle	94,466 atoms	12,110 atoms	83,622 atoms
Estimated percent surface atoms per particle	3.38%	13.0%	2.80%

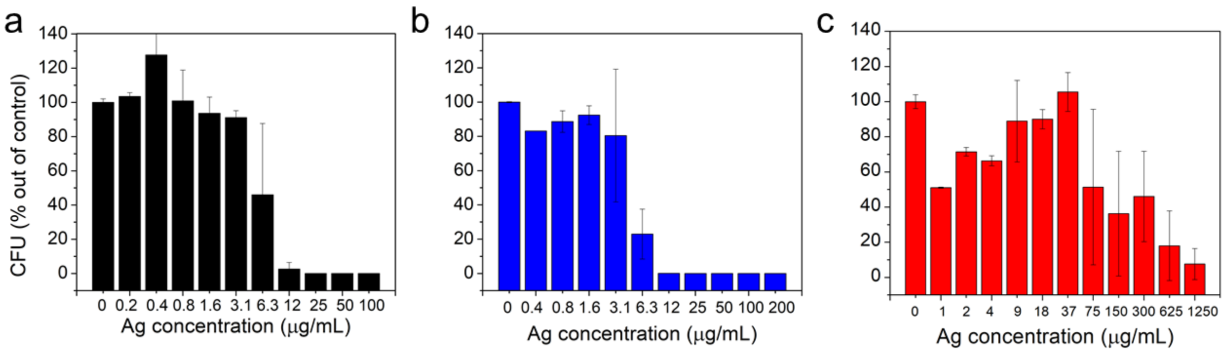

Figure S1. Antimicrobial activity of AgNPs to *E. coli*. (a-c) Viable CFU count after 3 h of contact time between AgNPs and *E. coli* K-12 using 0−1,60 μg/mL of the particles. All experiments are compared to the negative control (no AgNPs) (n = 9) with (a) corresponding to cube-AgNPs, (b) disc-AgNPs, and (c) pseudospherical-AgNPs.

Antimicrobial activity of AgNP shapes as a function of Ag(I) ion release

The contributions of the released Ag(I) ions to the observed antimicrobial activities of different shapes (**Figure S1**) were parsed by monitoring Ag(I) ion release at their respective EC_{50} concentrations in 0.9% NaCl over the exposure period (measured as total Ag in the supernatant). There is no difference in the *absolute quantity* of Ag(I) ions across the three shapes (**Figure 3D, S2A**). In other words, similar total amounts of Ag(I) ions are released from the particles at their EC_{50}. However, while there is the same amount of measurable Ag(I) ions at each shape's EC_{50}, the extent to which the particles dissolved are different since each shape's EC_{50} occurs at a different mass concentration (**Figure S1**) (i.e., the *percentage* of ion release across shapes is different). A greater percentage of ion release (~40x difference) is observed for cube- and disc-AgNPs compared to pseudospherical-AgNPs (**Figure S2B**), which aligns with the ~40x difference in their respective mass-based EC_{50} (pseudospherical-AgNPs have a much larger mass-based EC_{50}) (**Figure S1**). However, we cannot say if the Ag(I) ions alone are driving the differences in antimicrobial activity across shape (**Figure S1**) nor can we rule out the presence of other particle-specific factors contributing to the differences in antimicrobial activity. Because the relative antimicrobial activity shifts based on the dose metric (Figures 3, 4 in the main text), there is also an important particle parameter influencing antimicrobial activity. Thus, we cannot draw any conclusions surrounding the influence of Ag(I) ions on the differences in antimicrobial activity across shape. The mechanism may likely be a combination of both particle properties and Ag(I) ion release. A more robust set of experiments evaluating Ag(I) ion release is thus needed to clarify and unravel these competing mechanisms of shape-based antimicrobial activity.

Further, since disc-AgNPs have a greater percentage of surface atoms per particle (13.0% compared to 3.38% for the cube-AgNPs, **Table S1**) and a greater amount of total available surface

area at the EC_{50} as compared to the cubes – which is known to scale with dissolution (i.e., ion release relates directly to the amount of surface area available for interaction with oxygen) [5] – it is unexpected that these two shapes present similar ion release profiles. It is important to note that it was challenging to spin down the anisotropic shapes (i.e., the cube- and disc-AgNPs), which is the process that isolates the supernatant; the pellet would collect on the side rather than the bottom of the tube, so extra care was taken to withdraw the supernatant without disturbing the pellet. The disc-AgNPs were also subjected to an additional centrifuge spin to obtain a pellet. This may explain the high error bars for those shapes as compared to the pseudospherical-AgNPs as well as the variability in ion release over time.

The remaining particles in the pellet were then imaged with TEM after the 3-hr incubation period in 0.9% NaCl (**Figure S3**). The pseudospherical-AgNPs showed little degradation and retained similar size and shape which aligns with its Ag(I) ion release profile. Interestingly, the cube- and disc-AgNPs show noticeable degradation, the mechanism of which may not fully be explained by ionization alone as a low percentage of ionization occurs in each system (6.62% and 6.46% Ag(I) ion release for the cube-AgNPs and disc-AgNPs, respectively). Thus, ~94% of Ag remains in the system either in nanoparticulate form or as Ag chloride precipitate and so it is unexpected to observe such significant degradation. Ag(I) ion release was monitored at a single concentration (i.e., the EC_{50} concentrations associated with each shape) and represent vastly different total available surface areas. Monitoring Ag(I) ion release at multiple concentrations along the dose response curve and at similar total available surface areas would further elucidate the influence of Ag(I) ion release on both independent and relative trends in antimicrobial activity of AgNP shapes. Finally, this Ag(I) ion release study was conducted under the associated

experimental conditions (i.e., 0.9% NaCl); different experimental media will influence particle ion release profiles.

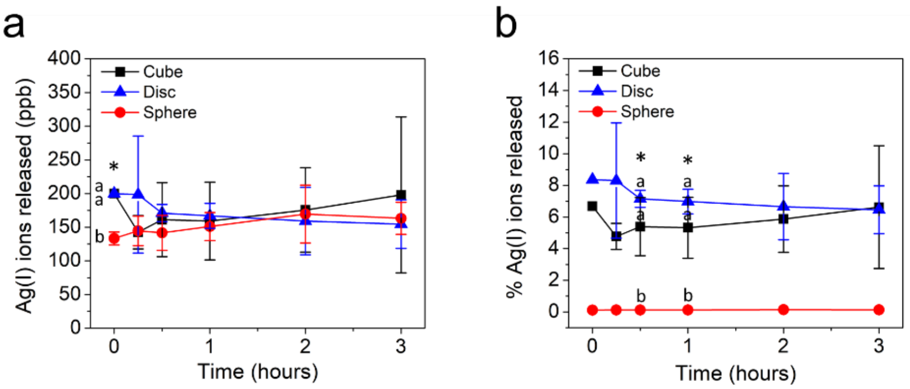

Figure S2. a) Total and b) percent Ag(I) ions released (measured by ICP-MS and ICP-OES) vs. time (at 0, 0.25, 0.5, 1, 2, and 3 h) for the AgNP shapes dispersed in 0.9% NaCl at 37 °C at their respective EC_{50} particle concentrations. Data points and error bars represent the average and standard deviation of two independent trials, with one being measured by ICP-MS and ICP-OES due to instrument availability during COVID-19 laboratory closures. Means suffixed with different letters (a–b) for each time point are significantly different from each other at $P < 0.05$.

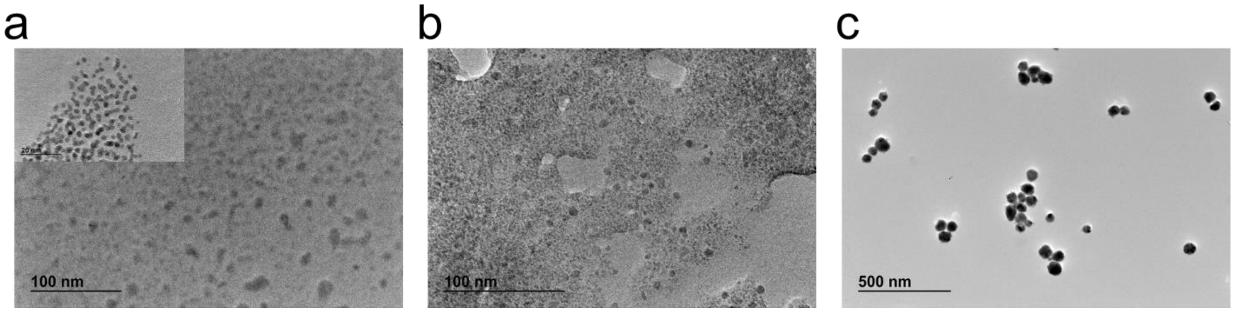

Figure S3. a) cube-, b), disc-, and c) pseudospherical-AgNPs after 3-hr incubation in 0.9% NaCl at 37°C at their respective EC_{50} particle concentrations, with the cube- and disc-AgNPs undergoing a degradation mechanism of unknown origin.

References

[1] Bastús, N.G., et al., *Synthesis of highly monodisperse citrate-stabilized silver nanoparticles of up to 200 nm: kinetic control and catalytic properties.* Chemistry of Materials, 2014. **26**(9): p. 2836-2846.

[2] Johnston, K.A., et al., *Impacts of broth chemistry on silver ion release, surface chemistry composition, and bacterial cytotoxicity of silver nanoparticles.* Environmental Science-Nano, 2018. **5**(2): p. 304-312.

[3] Siekkinen, A.R., et al., *Rapid synthesis of small silver nanocubes by mediating polyol reduction with a trace amount of sodium sulfide or sodium hydrosulfide.* Chemical physics letters, 2006. **432**(4-6): p. 491-496.

[4] Zhang, Q., et al., *A systematic study of the synthesis of silver nanoplates: is citrate a "magic" reagent?* Journal of the American Chemical Society, 2011. **133**(46): p. 18931-18939.

[5] Helmlinger, J., et al., *Silver nanoparticles with different size and shape: equal cytotoxicity, but different antibacterial effects.* RSC advances, 2016. **6**(22): p. 18490-18501.
